# Double-pivot proper digital artery perforator flap for fingertip reconstruction

**DOI:** 10.1186/s13018-023-04231-4

**Published:** 2023-09-28

**Authors:** Benquan Liu, Ding Pan, Zhiyu Gao, Pengfei Duan, Qifeng Ou

**Affiliations:** 1 Department of Hand Surgery, Zhoukou Orthopedic Hospital, Taihao Road, Chuanhui District, Zhoukou City, Henan China; 2 Present Address: Department of Orthopedics, Hand and Microsurgery, Xiangya Hospital, Central South University, No.87 XiangYa Road, Kaifu District, Changsha, Hunan China

**Keywords:** Fingertip, Reconstruction, Flaps, Proper digital artery, Perforator, Double-pivot design

## Abstract

**Background:**

Dorsal flap based on proper digital artery perforator has been commonly used in wound coverage of fingertip; yet a small diameter and short length poses a risk of pedicle kinking or occlusion. The present study aims to present our preliminary results of using a double-pivot perforator flap based on the end dorsal branch of proper digital artery to repair finger pulp defect.

**Methods:**

We designed a double-pivot flap based on the end-dorsal perforator branch of proper digital artery, raised from the dorsal aspect of the middle phalanx, with inclusion of both the perforator and a section of the trunk of the artery. This modified procedure forms a pedicle with a larger diameter and length than traditional designs. Twelve patients (12 fingers) each with a soft-tissue defect of the fingertip were successfully treated and followed up in this retrospective study.

**Results:**

All the flaps survived without showing any signs of necrosis; three cases presented with transient venous flow disorder, these self-resolving without requiring any additional treatment. At final follow-up (12–33 months, mean 20 months), mean static two-point discrimination on the flap was 7.0 mm (range, 6–9).

**Conclusion:**

The double-pivot proper digital artery flap serves as a reliable option in fingertip reconstruction offering added benefits of having greater rotation flexibility, a lower risk of vessel kinking or occlusion, and good recovery of cutaneous sensation.

**Supplementary Information:**

The online version contains supplementary material available at 10.1186/s13018-023-04231-4.

## Background

Soft-tissue injuries to the fingertips are common and may require reconstructive surgery with a flap. Over the last few decades, many different flap options have been introduced. Pedicled flap transfer includes the V–Y advancement flap [1], the reverse-flow flap [2], the cross-finger flap [3], the thenar flap [4]. However, each has disadvantages including limited distance of coverage, sacrifice of perfusion from the proper digital arteries, a long-period of immobilization, and all with potential flap survivorship-related complications. For instance, a dorsal flap on web space was adopted to successfully repair soft-tissue defect on thumb pulp and also on index finger when the flap is “cross-finger” [5]. However, as a pedunculated flap, the nonnegligible distance between donor site (web space) and fingertip necessitates a lengthy incision, and resultantly a long scar. Although there was no reported complication like narrowed web space, it might be technically demanding to minimizing the impact to zero. Free toe flap transfer is ideal for fingertip reconstruction for an extra benefit of restoring the contour of nail and concealed donor site morbidities [6]. Nevertheless, the related microsurgical anastomosis of perforators is time-consuming and technically demanding.

To date, the dorsal flap based on the proper digital artery (PDA) perforator has been commonly used in fingertip reconstruction. However, partial or total flap loss is not uncommon. For instance, 3 out of 7 patients were reported to experience flap necrosis with respective proportions of 30%, 40% and 80% [7]. In a review of 166 cases by Chen et al. [9], venous congestion was seen in 18 patients (10%), and partial flap necrosis was observed in 14 patients (8%) [8]. A small case series reported the incidence rate of partial necrosis is 9% [9].

Anatomically, the PDA perforator is the thinnest perforator (0.2–0.5 mm) [10] in the human body, and is also of short length (0.8–1.2 cm). Based on these anatomical features, in a 90°–180° rotation, the effective stress on the pedicle would be significant [11]. Consequently, the perfusion-related problems including vascular compromise, flap necrosis and cold intolerance are hard-to-avoid. Also, the short pedicle poses an obstacle in flap rotation, limiting the distance of the defect for which it can be used.

The trunk of PDA, however, has a diameter ranging between 0.8 and 1.3 mm, considerably greater than the perforator branches. In nonlinear finite element simulations of pedicle flaps, it has also been found that the minimum strain could be achieved on a 1-mm-diameter vessel [11]. This hemodynamic evidence indicates a better chance of patency of the PDA trunk than the perforator branch. Therefore, we introduced a PDA trunk segment in addition to the end-dorsal branch within the pedicle. In this way, the main pivot point is shifted from a point of thin diameter on the perforator branch, to a point of larger-diameter on the trunk of the PDA, so lowering risk of pedicle kinking or occlusion. Additionally, the length of pedicle is increased and the pivot axis is doubled, allowing more flexible transfer to a distant area. This study describes the technique and initial experience.

## Methods

From May 2017 to January 2020, twelve patients (12 fingers) with soft-tissue damage or amputated distal phalanges due to hand injury were treated with our digital artery perforator flap and included in this retrospective study. All of the operations were conducted in one-stage by flap coverage immediately after the radical debridement. This study conforms to of the guidelines of the Medical Ethics Committee of Zhoukou Orthopedic Hospital, the Helsinki Declaration in 1975 and its subsequent amendments; and informed consent was obtained from every patient prior to conducting operation.

### Patients

Of these twelve patients, there were eight male and four female with mean age of 41 years (range 22–63) (Table 1). Among these twelve cases, nine cases were caused by stamping press injuries, one cases by motor-driven saw, and two cases were crush contusion by large-weight items. Four cases involved the index finger, seven the middle finger and one the ring finger. The soft tissue defect sizes ranged from 1.2 × 1.6 cm to 2.0 × 2.8 cm, with a mean area of 4.1 cm^2^. All were located on the fingertips with bone exposure. There were no obvious injuries between the distal phalanx and the proximal phalanx. Five defects were located volar oblique on the fingertip, two defects transversely, and five were dorsal oblique. Eight of the cases were single finger injury and four cases included injury to multiple fingers. Mean time from injury to surgery was 6.3 h (3.8–10). After the surgery, papaverine (30 mg/times, 2–3 times/day) was used as an anti-coagulant routinely for those patients. And for elderly patients (> 60 years old) or those with a high risk of thrombus, we used low molecular weight heparin as a replacement.

**Table 1:** Patient information

Patients	Age (years)	Sex	Mechanism of injury	Injured finger	Defect	Length of terminal perforator (cm)	Length of disassociated PDA trunk (cm)	Flap dimension (cm)	Vascular compromise	2PD (mm)	Follow-up (months)
1	42	F	Stamping press	Right middle	Volar oblique	0.8	1.7	1.5 × 1.8	None	8	14
2	63	M	Stamping press	Right ring	Dorsal oblique	1.0	1.6	1.6 × 2.2	None	6	12
3	33	F	Stamping press	Right middle	Transverse	0.9	1.9	1.6 × 2.5	None	6	20
4	28	M	Stamping press	Left middle	Volar oblique	1.2	1.6	2.2 × 3.2	Transient tension blister	8	33
5	46	M	Stamping press	Right middle	Volar oblique	1.3	1.5	1.8 × 2.3	None	7	31
6	26	M	Stamping press	Left index	Transverse	1.0	1.6	1.5 × 1.8	None	6	17
7	31	M	Stamping press	Right index	Volar oblique	0.9	1.7	1.4 × 2.2	Transient tension blister	6	22
8	28	M	Stamping press	Left middle	Dorsal oblique	1.1	1.6	1.6 × 2.4	None	7	10
9	22	F	Stamping press	Right middle	Dorsal oblique	1.0	1.5	2.0 × 2.8	None	6	25
10	48	M	Motor-driven saw	Left middle	Dorsal oblique	0.8	1.6	1.6 × 2.4	None	7	15
11	52	M	Crush contusion	Right index	Volar oblique	1.1	1.9	1.5 × 2.6	None	8	18
12	31	F	Crush contusion	Left index	Dorsal oblique	1.2	1.5	1.7 × 2.5	Transient dark purpled flap	9	22

2PD—Two point discrimination; PDA—proper digital artery. M—male; F—female

### Flap design

**Fig. 1:**
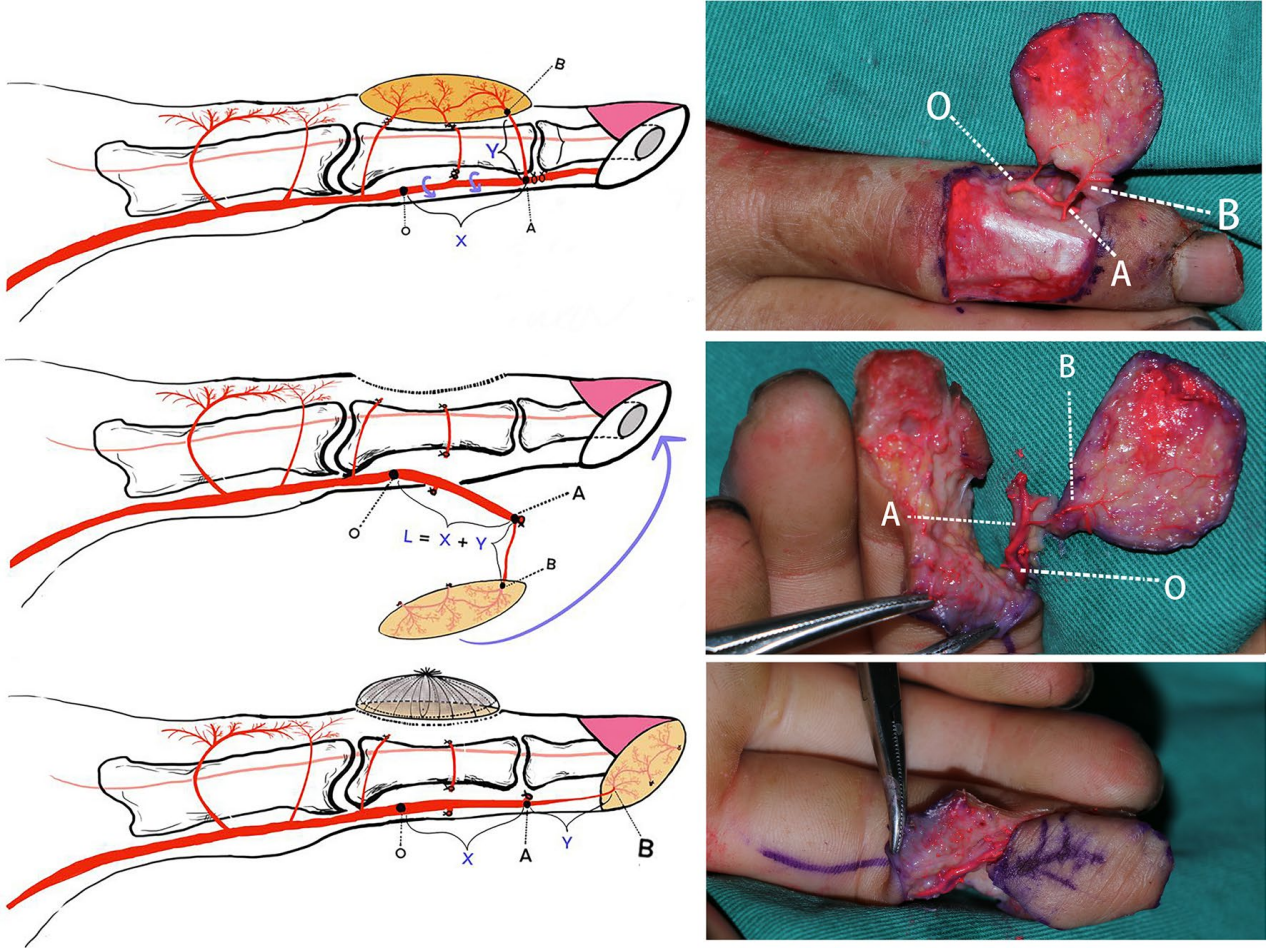
This Figure presents a schematic illustration (Left) and a cadaver dissection (Right) of a double-pivot proper digital artery perforator flap to reconstruct the soft-tissue defect on distal phalanx. X + Y = The total length of the pedicle (O–A–B). X = length of the disassociated main trunk of the proper digital artery (O–A). Y = length of the end-dorsal perforator branch (A–B). The points O, A and B respectively refer to the main pivot point on the PDA trunk, the second pivot point where the end-dorsal perforator originates from the PDA, and the penetrating point on the flap. The flap is extended proximally by preserving the vessel chain between end-dorsal perforator and proximal perforators. The donor site on the middle phalanx is covered by a full-thickness skin graft

The pedicled flap was designed from the dorsal aspect of the middle phalanx (Fig. 1) selecting either the radial or ulnar proper digital artery depending on which one was closest to the edge of the wound. The flap did not extend past the mid-lateral line nor distally past the distal interphalangeal joint, thereby avoiding any dermatoglyphic injury that may compromise the mobility of the joint. The pedicle was designed using two continuous segments, the double-pivot axes formed by two-pivot points (Fig. 1). The main pivot axis refers to the PDA trunk with a customized length to permit a necessary flexibility in rotating the flap, while the second pivot axis refers to the entire course of the end-dorsal branch. By doing this, in this flap, the main pivot point is on PDA trunk, and second pivot point is located at where end-dorsal branch emanates from PDA trunk. This pedicle design consisting of a main pivot point in addition to a second pivot point is similar to the “double-pivot” design previously described by Karacalar [12] for the second dorsal metacarpal artery flap. Additionally, by preserving the vessel chain between end-dorsal perforator and proximal perforators, the flap was extended proximally, which has been well recognized and illustrated in previous literature [10].

When harvesting the flap, the lateral margin of the flap was first incised, extending all the way to the wound edge. The skin was dissected off the subcutaneous fat layer and lifted up to expose the terminal segment of the PDA, as well as, the corresponding nerve and its stump. During this process, the second pivot point (Fig. 1A) where the end-dorsal perforator branches from the proper digital artery could be clearly identified on the deep surface of Cleland’s ligament. Thereafter, from the branching point, the end-dorsal branch was dissected dorsally along its course around the distal interphalangeal joint (Fig. 1) until the penetrating point was reached. Following this, we incised the remaining three edges of the flap, beneath which the penetrating point could be re-identified.

At that point, the flap and the second pivot axis had successfully been obtained. Subsequently, returning to the lateral margin, further dissection was conducted to obtain a segment of the proper digital neurovascular bundle to serve as the main pivot axis. To do this, the distal end of the PDA trunk was ligated; and the pedicle was extended from end-dorsal perforator branch part (second pivot axis) to proximal part of PDA (first pivot axis) by meticulously disassociating PDA trunk. As the anatomic dissection of the neurovascular pedicle is performed on the PDA trunk, the perforator branches remain within the fascial tissue (usually 0.5 mm in thickness) and not dissected. The PDA trunk should not be overly naked either, in case of unnecessary damage to concomitant vein and PDN. It is noteworthy that the length of the trunk should be adjusted to allow a sufficient degree of freedom, which can be assessed by observing abundant bleeding on the flap edge. Thereafter, the flap, was rotated around the main pivot point to the wound (Point O, Fig. 1). Lastly, the donor site on the dorsal middle phalanx was covered with a full-thickness skin graft harvested from the medial side of the upper arm.

### Outcome evaluation

Complications including vascular compromise, flap loss, cold intolerance, flexion contracture and skin pigmentation were recorded. Sensation (static two-point discrimination) was evaluated using static two-point discrimination by the device (Baseline, touch-test, 12-1495).

## Results

**Fig. 2:**
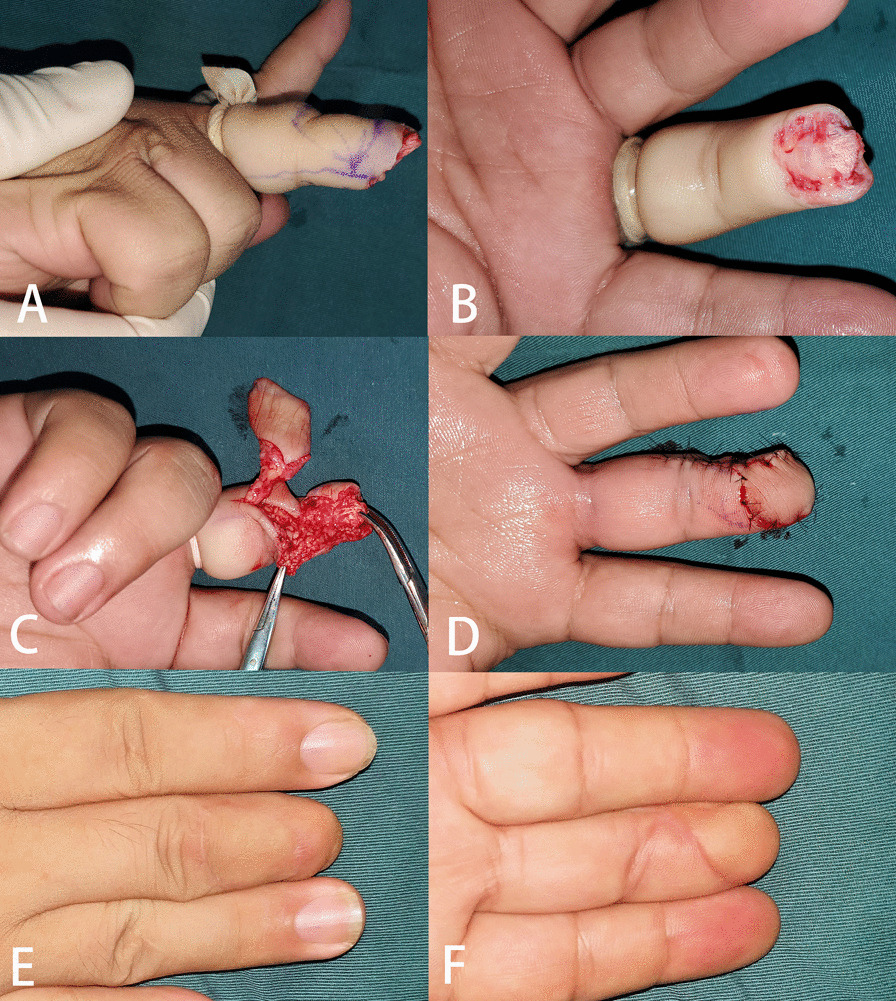
Case 1. Reconstruction of a volar oblique defect by a double-pivot proper digital artery flap; **A** and **B** show the soft-tissue defect and also the donor site for the flap (blue lines in **A**); **C** and **D** refer to the flap transfer and (**C**) coverage (**D**); **E** and **F** refer to the postoperative appearance of the donor site and reconstructed area

**Fig. 3:**
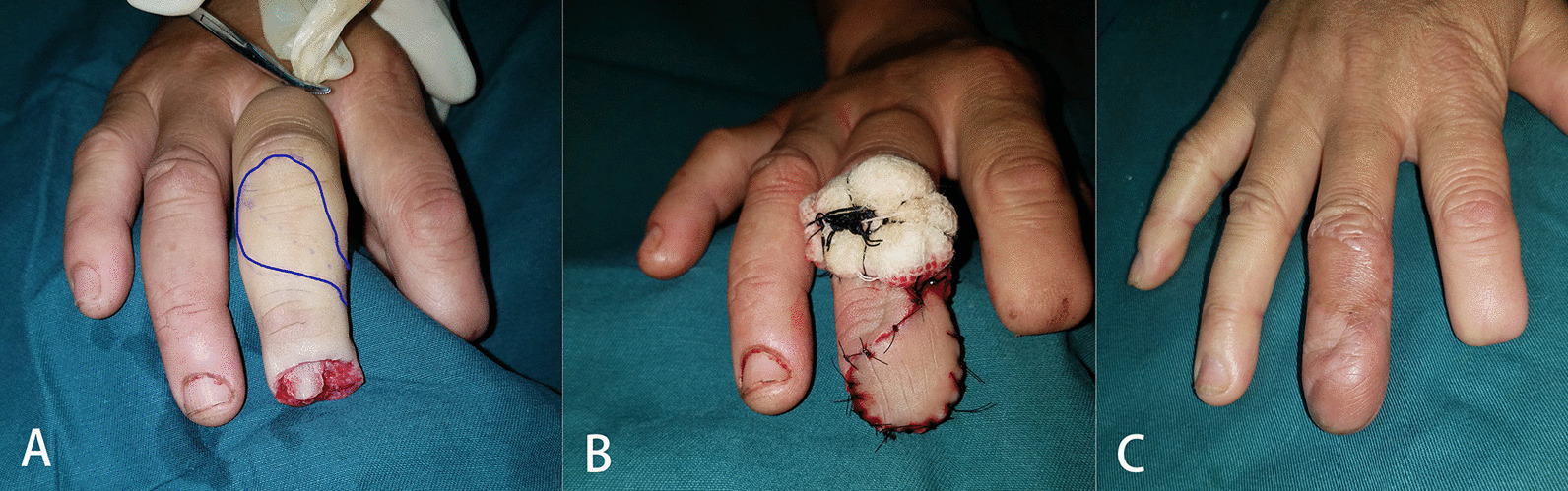
Case 3. A transverse amputation of the distal phalanx (**A**) was reconstructed using the double-pivot proper digital artery flap (**B**). The flap is marked before the dissection (**A**, marked by blue stroke). Postoperatively, the flap survived well without skin pigmentation (**C**)

All the flaps survived and the incisions healed after one-stage reconstruction. Neither total nor partial flap loss was observed; and in only three cases did postoperative venous disorder appear, of which two presented tension blisters, and one presented dark purple color. They were transient and resolved without requiring any special treatment. The flaps survived well with good skin color (Figs. 2, 3 and Additional file 1: Fig. S1), and the skin graft did not cause any contracture during the follow-up. Other complications such as cold intolerance, and flexion contracture did not occur. At final follow-up (mean 20 months, range 10–33), mean static two-point discrimination on the flap was 7.0 mm (range, 6–9).

## Discussion

In this study, the double-pivot design enabled the PDA perforator flap to be successfully transferred to the fingertip defects. The flap was based on the end-dorsal perforator branch, and the defects were transverse or oblique (dorsal or volar). No flap loss or major vascular complications occurred. Previously, a number of perforator-based homo-digital flaps have been reported. Primarily introduced by Koshima [13], it has developed into several variants including adipose-only flap, adipo-cutaneous flap, supercharged flap [14], and an extended flap which was harvested by preserving the links between the perforator with perforators [10] or with the dorsal digital artery [15]. However, the pivot axis was always positioned on a perforator of thin diameter and short length. Although the flap necrosis rate is not considered to be high, neither is it negligible, and thus worth attention in trying to find better ways of countering it. We believe this double-pivot flap is more likely to survive, and should be considered especially when perfusion or outflow is normal after flap elevation but compromised after pedicle rotation.

Besides our approach, numerous simpler techniques for fingertip reconstruction have been documented in previous publications [16]. The V–Y advancement flap has a minimized technical demands owing to minimized necessities for artery disassociation, and as a results, a decreased surgical time and less technical demands; yet it is more preferred in 0.3–0.5 mm defects [17], whereas the lengths of defects in the present study ranged 1.5–2.2 cm. Similar as V–Y advancement, parallelogram flaps, which maintain technical simplicity, are empowered with an enhanced capacity of coverage in size; however, the advanced distance is maximally up to 6–10 mm [18]. Pivot flaps can cover palmar defects 1.8–2.5 cm long, and still maintain the technical simplicity [19]. Nevertheless, it cannot be transferred to a dorsal defect, and the potential risk of creating a hook nail deformity should be appreciated by both the surgeon and the patients. Recently, the moist antiseptic dressings have received global use [20, 21], some artificial dermal substitute could even circumvent the necessity of skin graft and yield satisfying aesthetic outcome [22]. However, the usage of these substitute on bone exposure is still controversial [23], while this exposure was present in most of the cases in present study; an option is nibbling the protruding bone, which yet creates a shortened finger length [24]. But we do agree that this less invasive approach is promising and deserving more appreciation in repairing finger pulp. Additionally, we did not consider the free toe flap, because we think the microsurgical anastomosis is time-consuming and technical-demanding. Admittedly, free toe flap are fabulous choice if the defect exceeds 3 × 3 cm in size [25]. Of notes, selecting an optimal approach to reconstruct soft tissue on fingertip should consider the size and numbers of defects, availability of skin on donor sites, expectation from patients, and expertise of surgeons.

The main advantage of our double-pivot flap design lies in a flexibility in transposition of flap, by a large diameter in pivot point lowering the risk of vessel kinking or occlusion. An additional benefit is that the double-pivot design removes the need for accurate dissection of the perforator vessel. When raising the flap, the anatomic dissection of the neurovascular pedicle is performed on the PDA trunk, while the perforator branches remain safely intact within the fascial tissue thus saving time and reducing the overall level of difficulty.

It is also worth to mention that the proper digital nerve (PDN) remains attached within the neurovascular bundle when the PDA trunk is disassociated. Nevertheless, our flap was not innervated by dorsal digital nerve (DDN). The dorsum of middle phalanx, of which our flap was based, is anatomically dominated by mainly DDN and in part by dorsal branches of proper digital nerves (DBPDNs) [26]. Admittedly, a coaptation of DDN from flap to collateral proper digital artery might increase the possibility of enhanced or accelerated sensation recovery. Yet outcome of static two-point discrimination in present study ranged from 6 to 9 mm, as acceptable as those of many other studies using innervated homo-digital flap. Part of the reason might be the proper digital nerve trunk was harvested along with the PDA trunk, to be included as part of the pedicle, and possibly a proportion of DBPDN traveling with the end-dorsal perforator was included in the vascular pedicle as well.

The following are some technical points to help ensure a successful operation. First, before the flap is designed, it should be made certain that the end-dorsal perforator has not been contused by the injury. Otherwise, vascular compromise or flap necrosis may occur. Second, in the operation it is not required for the end-dorsal perforator to be fully dissected or exposed and avoiding doing this will help prevent damage to the concomitant vein and nerve. Third, along the tunnel for pedicle placement, the subcutaneous fascia tissue should be released or removed if necessary, to allow an appropriate degree of looseness and thereby avoid potential compression on the pedicle.

In conclusion, the double-pivot proper digital artery perforator flap serves as a reliable option in fingertip reconstruction. By harvesting a segment of the PDA trunk for the pedicle, an additional length and a larger-diameter pivot axis are obtained thereby allowing for greater flap rotation flexibility, a lower risk of vessel kinking or occlusion, and a better recovery of sensation. Further large-scale study is still needed to define the indications and contraindications of this technique.

### Supplementary Information

**Additional file 1: Figure S1** Case 9. A dorsal oblique defect (**A**) on the distal phalanx was reconstructed using a double-pivot proper digital artery flap (blue line, **B**). Donor-site was covered by a skin graft under compression (**C**). Postoperatively, the flap survived well without skin pigmentation (**D**).
