# Supplementary material for: Double-pivot proper digital artery perforator flap for fingertip reconstruction
Source: J Orthop Surg Res. 2023 Sep 28;18:737. doi: 10.1186/s13018-023-04231-4 (PMC10540400; doi:10.1186/s13018-023-04231-4)
Supplement: Supplementary file 1 — Additional file 1: Figure S1 Case 9. A dorsal oblique defect (A) on the distal phalanx was reconstructed using a double-pivot proper digital artery flap (blue line, B). Donor-site was covered by a skin graft under compression (C). Postoperatively, the flap survived well without skin pigmentation (D). [file 13018_2023_4231_MOESM1_ESM.docx]

Supplementary material:

Figure S1


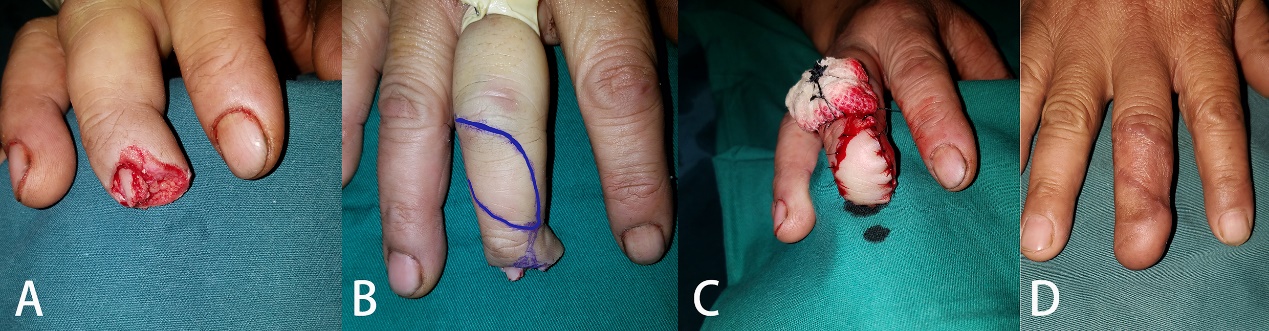


Figure S1. Case 9. A dorsal oblique defect (A) on the distal phalanx was reconstructed using a double-pivot proper digital artery flap (blue line, B). Donor-site was covered by a skin graft under compression (C). Postoperatively, the flap survived well without skin pigmentation (D).
